# FluoroPi Device With SmartProbes: A Frugal Point-of-Care System for Fluorescent Detection of Bacteria From a Pre-Clinical Model of Microbial Keratitis

**DOI:** 10.1167/tvst.12.7.1

**Published:** 2023-07-03

**Authors:** Syam Mohan P. C. Mohanan, Kay Russell, Sheelagh Duncan, Alex Kiang, Charles Lochenie, Emma Duffy, Stephnie Kennedy, N. Venkatesh Prajna, Rachel L. Williams, Kevin Dhaliwal, Gareth O. S. Williams, Bethany Mills

**Affiliations:** 1 Translational Healthcare Technologies Group, Centre for Inflammation Research, University of Edinburgh, Edinburgh, UK; 2 Department of Eye and Vision Science, University of Liverpool, Liverpool, UK; 3 Department of Cornea and Refractive Surgery, Aravind Eye Hospital, Madurai, India

**Keywords:** microbial keratitis, optical imaging, low cost, diagnostic, fluorescence

## Abstract

**Purpose:**

Rapid and accurate diagnosis of microbial keratitis (MK) could greatly improve patient outcomes. Here, we present the development of a rapid, accessible multicolour fluorescence imaging device (FluoroPi) and evaluate its performance in combination with fluorescent optical reporters (SmartProbes) to distinguish bacterial Gram status. Furthermore, we show feasibility by imaging samples obtained by corneal scrape and minimally invasive corneal impression membrane (CIM) from ex vivo porcine corneal MK models.

**Methods:**

FluoroPi was built using a Raspberry Pi single-board computer and camera, light-emitting-diodes (LEDs), and filters for white-light and fluorescent imaging, with excitation and detection of bacterial optical SmartProbes: Gram-negative, NBD-PMX (ex_max_ 488 nm); Gram positive, Merocy-Van (ex_max_ 590 nm). We evaluated FluoroPi with bacteria (*Pseudomonas aeruginosa* and *Staphylococcus aureus*) isolated from ex vivo porcine corneal models of MK by scrape (needle) and CIM with the SmartProbes.

**Results:**

FluoroPi provides <1 µm resolution and was able to readily distinguish bacteria isolated from ex vivo models of MK from tissue debris when combined with SmartProbes, retrieved by both scrape and CIM. Single bacteria could be resolved within the field of view, with limits of detection demonstrated as 10^3^ to 10^4^ CFU/mL. Sample preparation prior to imaging was minimal (wash-free), and imaging and postprocessing with FluoroPi were straightforward, confirming ease of use.

**Conclusions:**

FluoroPi coupled with SmartProbes provides effective, low-cost bacterial imaging, delineating Gram-negative and Gram-positive bacteria directly sampled from a preclinical model of MK.

**Translational Relevance:**

This study provides a crucial stepping stone toward clinical translation of a rapid, minimally invasive diagnostic approach for MK.

## Introduction

Microbial keratitis (MK) caused by bacteria or fungi remains a significant cause of blindness and visual complications globally.^1^^–^^5^ Rapid and accurate diagnosis of the causative microorganism is critical for implementation of appropriate treatment strategies with antibiotics or antifungals. Current gold-standard diagnostic methodology involves culturing of corneal scrapes, which has a reported positivity rate of only 50%.^3^^,^^6^ Combined with lengthy incubation times (2–14 days), this renders diagnostic cultures as somewhat impractical for rational prescribing of drugs for this ophthalmic emergency. Direct smear microscopy examination to identify the presence of fungi or elucidate the Gram status of bacteria can provide a same-day result, but success rates are highly variable among centers, with pathogens identified between 27.3% and 61.6% of the time.^3^ There is therefore an important and burgeoning need to improve rapid diagnosis of MK.

We have previously reported on the utility of fluorescent reporters (SmartProbes) to rapidly identify bacteria and fungi directly from corneal scrapes from a patient population in south India using a single-channel commercial microscope. We found that sensitivity, specificity, negative predictive value, positive predictive value, and accuracy were equivalent to or better at matching gold-standard culture results compared to conventional Gram stain.^7^ However, several technical constraints limit the broad translational potential of this approach. The first is the requirement of a high-resolution fluorescence microscope capable of multiple excitation lines for the multiplexed detection of exogenous fluorophores from a single sample. These are prohibitively expensive in many clinical scenarios and require technical expertise to operate and maintain. The second limitation is the reliance on clinical scrapes to collect microbial isolates for interrogation (by any diagnostic methodology), thus confining the diagnosis of MK to specialist tertiary care facilities. Less invasive sampling techniques, such as the utilization of a corneal impression membrane (CIM),^8^^,^^9^ may one day enable specimen collection to be devolved toward secondary and primary care centers by allied healthcare professionals, thus increasing patient convenience and promoting earlier engagement with the healthcare system when infections are more likely to have a favorable clinical outcome.^2^^,^^3^^,^^8^^,^^10^^–^^13^

We have sought to address these limitations through the development of a proof-of-concept, low-cost (comparable price point to a cell phone), dual-channel fluorescence imaging device (FluoroPi) which further combines white-light morphological imaging. The two main approaches to producing low-cost fluorescent imaging devices have been the use of a smartphone as the basis of detection^14^^–^^18^ and bespoke devices based on single-board computers.^19^^–^^22^ We chose this latter approach to avoid inter-phone compatibility challenges (e.g., camera specifications, requirement of bespoke adaptors, mounts, and software for each make and model), which is a major limitation associated with smartphone-based systems. Our FluoroPi device utilizes a Raspberry Pi single-board computer and camera, inexpensive coupling optics, and multiple light-emitting-diode (LED) sources and filters to allow for straightforward fluorescent imaging of multiple fluorophores requiring only a simple, user-accessible filter swap. We have demonstrated its application in identifying and distinguishing Gram-negative and Gram-positive bacteria (exemplified by *Pseudomonas aeruginosa* and *Staphylococcus aureus*, respectively) isolated from corneal scrapes of ex vivo porcine models of MK utilizing fluorescent optical reporters 7-nitrobenz-2-oxa-1,3-diazole (NBD)-polymyxin (PMX) (ex_max_ 488 nm)^23^ for Gram-negative detection, and merocyanine (Merocy)-vancomcyin (Van) (ex_max_ 590 nm)^24^ for Gram-positive detection. The NBD-PMX binding domain is a derivative of polymyxin B and specifically attaches the SmartProbe to lipid A on the cell surface of Gram-negative bacteria, whereas Merocy-Van utilizes a derivative of vancomycin as its binding domain and therefore anchors the SmartProbe within the exposed peptidoglycan of the Gram-positive cell wall. These SmartProbes each have a spectrally distinct, environmentally activated fluorophore that only fluoresces when the ligand–fluorophore conjugate is in close proximity to the hydrophobic cell envelope, producing high signal to noise for wash-free imaging. These SmartProbes have been extensively characterized and described in previous studies.^23^^,^^24^ Here, we compare the resulting FluoroPi images to a commercial microscope system (DMi8 Basic; Leica Microsystems, Wetzlar, Germany) to evaluate their comparative performance.

We have further explored the incorporation of a CIM with our ex vivo porcine MK models. The CIM has recently been introduced into routine clinical practice at the St. Paul's Eye Unit (Liverpool, UK) and demonstrated significantly improved culture positivity rates compared to traditional scraping methodologies, attributed to enhanced sampling.^8^^,^^9^ We coupled the CIM collection method with SmartProbe interrogation and a FluoroPi imaging device as a first step toward less invasive sampling followed by simple and rapid diagnostics of MK.

## Materials and Methods

### FluoroPi Device Details and Characterization

An overview of the developed FluoroPi device is given in the Results section. Details of the image capture and processing methods are provided below, and the full FluoroPi device details, image postprocessing, and technical characterization, including resolution and field of view (FoV) determination, are outlined in the Supplementary Information.

### Image Capture With Commercial Microscope

The samples were imaged with a commercial widefield imaging system (Leica DMI8 Basic), with bright-field, fluorescein isothiocyanate (FITC)-cube, and Texas Red–cube imaging parameters selected (referred to as blue LED and orange LED, respectively, within the text) and a 40× air objective. Three FoVs per sample were collected. Imaging parameters were kept consistent within experimental repeats. Images captured with the commercial system were processed with Leica LAS X software and cropped to match the imaging FoV of FluoroPi. Brightness and contrast adjustments were consistent across datasets.

### Image Capture With FluoroPi

The FluoroPi system can be operated in several imaging modes. Operating the camera via open-source Python scripts (https://picamera.readthedocs.io/en/release-1.13/)^25^ enables changes to exposure time and ISO settings. A preview mode allowed for real-time imaging and optimization of focus, as well as positioning of the FoV, before subsequent image capture with bright-field and fluorescence modes. For bright-field imaging, a low exposure time and ISO mode were used with the white-light LED, whereas for low-light fluorescence imaging a long exposure and noise-optimized mode was used. Parameters used for image acquisition are given in the Table and were consistent for all samples and fluorescence excitation colors. Prior to sample image capture, a blank slide containing equimolar concentrations of SmartProbe used within the study was captured using the same imaging parameters to account for any background fluorescence emission and to enable background subtraction during postprocessing.

**Table.** Imaging Parameters for White-Light and Fluorescence Image Capture

Camera Parameters	White-Light Image	Fluorescence Image
Exposure time	200 µs	9 s
ISO	43^a^	800
Frame rate (frames per second)	—^b^	1/6

a Default camera value.

b Automatically adjusted by camera software based on available resources.

For each slide, the regions of interest were chosen by scanning over the sample using the *x*-*y* adjusters on the stage to find a suitable FoV (identified by the presence of a biomaterial/morphological feature, such as undistinguishable debris or bacteria, within the field of view by white-light imaging). This was done using the preview imaging mode of the system. Captured images were stored on the Raspberry Pi camera and subsequently transferred to a personal computer (PC) for postprocessing. At least three images were captured per slide. All imaging sessions were repeated independently at least three times.

### Fluorescent Bead Sample Preparation

Commercially available fluorescent beads were utilized to test the performance of the device. The beads included (1) Inspek green fluorescent beads (3% intensity and 2.5 µm in size, I7219; Invitrogen, Waltham, MA), and (2) fluorescent purple particles (medium intensity and 1.7–2.4 µm in size, FP-2062-2; Spherotech, Lake Forest, IL). The bead stock solutions were vortexed prior to dilution 1:10 in sterile saline (Baxter, Deerfield, IL). Then, 20 µL of the diluted samples was added to glass slides, mounted with a coverslip, and sealed with clear nail polish. The beads were added to slides independently and in combination for individual and dual samples, respectively.

### Bacterial Sample Preparation

The Gram-negative bacteria *Pseudomonas aeruginosa* PA01 and Gram-positive bacteria *Staphylococcus aureus* ATCC25923 (planktonic bacteria experiments) and *S. aureus* IHMA2190153 (ex vivo porcine model experiments) were utilized for this study as representative Gram-negative and Gram-positive bacteria. Single colonies were picked from fresh Luria-Bertani (LB) agar plates and were used to inoculate 10 mL LB broth; they were grown for 16 hours with shaking (250 rpm) at 37°C. The cultures were diluted to optical density at 600 nm (OD_600_) = 0.1 and grown to mid-log phase (∼OD_600_ = 0.7). An OD_600_ of 1 (equivalent to 10^8^ colony-forming units (CFU)/mL) for each strain was harvested and washed in sterile saline (Baxter) with microcentrifugation at 13,000 rpm for 1 minute. The resultant pellet was resuspended in 1 mL sterile saline. As required, bacterial samples were serially diluted 1:10 in saline within a 96-well plate to prepare bacterial samples for limit of detection testing and plating of CFU.

The NBD-PMX and Merocy-Van SmartProbes were manufactured in-house following reported protocols^23^^,^^24^ and were added to the bacteria as appropriate at the following final concentrations: NBD-PMX (2.5 µM) and/or Merocy-Van (1 µM). Then, 20 µL of each sample was placed on a standard glass slide, mounted with a coverslip, and sealed with clear nail polish prior to imaging. All experimentation was completed independently three times.

### Ex Vivo Porcine Cornea Preparation and Infection

Fresh porcine eyes were retrieved from pigs culled at a local abattoir. Ex vivo porcine cornea (*n* = 18) were excised and prepared based on methods previously described.^26^^,^^27^ Further details are provided in the Supplementary Information. Bacteria for cornea infection were prepared as described above. The corneal epithelium was removed by scraping with a scalpel blade, and 10 µL of the *P. aeruginosa* or *S. aureus* suspension or vehicle control was seeded onto the surface of the central cornea by pipetting and allowed to dry onto the cornea surface (*n* = 6 per group). Then, 3 mL of Dulbecco's modified Eagle's medium (DMEM, 10% [v/v] fetal bovine serum (FBS)) was added to the well with care taken not to wet the cornea. The corneas were incubated at 37°C for 18 hours, at which point corneal defects (clouding) indicating ulceration and established infections were visible (as shown later in Fig. 4A).

### Ex Vivo Corneal Infection Sampling

After overnight incubation, the cornea were sampled by two methods: corneal scrape (*n* = 3 per group) or CIM (0.4-µm pore size, hydrophilic polytetrafluoroethylene (PTFE), *n* = 3 per group; MilliporeSigma, Burlington, MA). The visibly infected area of the cornea was scraped using a needle (23-gauge BD Microlance; Becton Dickinson, Franklin Lakes, NJ) to mimic clinical specimen retrieval, applied directly to a glass slide, and left to air dry. Alternatively, the infected area was sampled using a CIM by gently placing the CIM onto the cornea for 5 seconds. Then, 100 µL sterile saline was added to the CIM, mixed with the sample, and then transferred to an Eppendorf tube. Of this, 20 µL was added to a glass slide and left to air dry; 20 µL of 2.5 µM NBD-PMX^23^ and 1 µM Merocy-Van^24^ in saline was then added to the sample, which was mounted with a coverslip sealed with clear nail polish. The prepared slides were subsequently imaged with FluoroPi and a commercial widefield imaging system.

Following sample collection, the center of each cornea was extracted by 8-mm biopsy punch and homogenized (Precellys 24 tissue homogenizer; Bertin Instruments, Montigny-le-bretonneux, Ile-de-France, France). Homogenized tissue samples and CIM samples were plated onto LB agar and incubated at 37°C overnight. *P. aeruginosa* and/or *S. aureus* growth was confirmed.

## Results

### FluoroPi System

**Figure 1.**
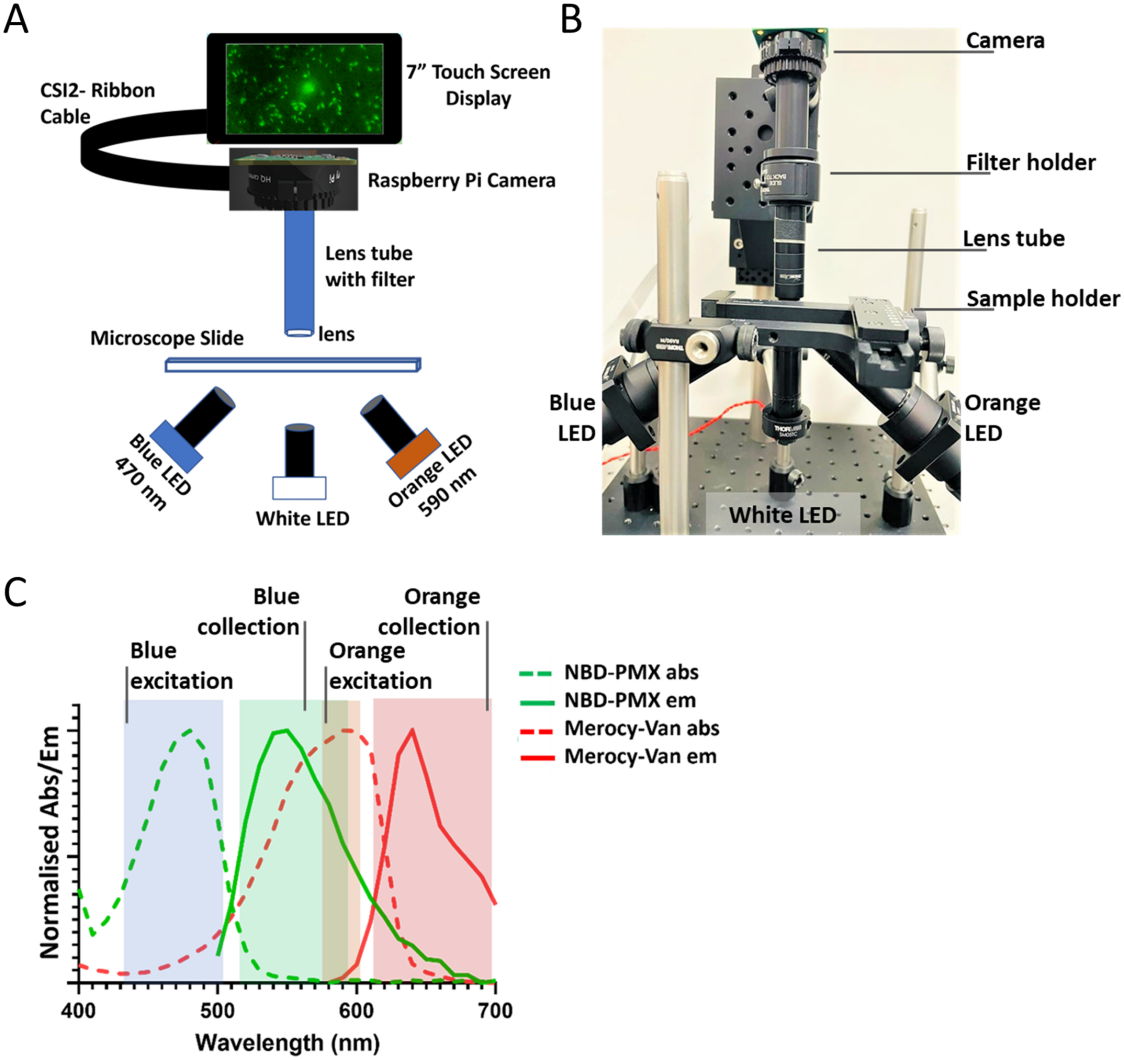
(**A**) Schematic of the fluorescence imaging setup (FluoroPi). (**B**) Photograph of the FluoroPi system. (**C**) Excitation (*dashed lines*) and emission (*solid lines*) spectra of SmartProbes: NBD-PMX (*green*) and Merocy-Van (*red*). *Solid-color* *blocks* show the FluoroPi blue and orange LEDs and associated emission filter band widths for fluorescence excitation and collection.

The FluoroPi device (Figs. 1A, 1B) consists of two main modules: (1) a detection module containing a camera and associated lens for image capture, and (2) an illumination module consisting of multiple LEDs and lenses for sample illumination and excitation. The illumination module LEDs consisted of a single white-light emitting LED for morphological imaging, and blue (470 nm) and orange (590 nm) excitation LEDs for fluorescent imaging. The wavelengths of these were chosen to match the target bacterial optical reporters, the excitation and collection bands of which are shown in Figure 1C and Supplementary Figure S1. The emission filter (used to remove any remaining excitation light) in the detection module was easily interchangeable based on the illumination LEDs in use, with no change to the rest of the optical setup required, or need for realignment. This enabled rapid sequential imaging of multiple reporters without the need for sample adjustment or the need for a costly multiband filter. There is a microscopy slide holder for sample placement and adjustment, as well as a Raspberry Pi single-board computer^28^ with associated touch screen for user input. The Raspberry Pi camera was used for both the fluorescence and white-light image acquisition. Python scripts were used to drive the camera system, with further postprocessing applied offline (see the Materials and Methods section and Supplementary Information).

The resolution was found to be ∼630 nm by performing a Gaussian fit to a line profile of the smallest features of a United States Air Force (USAF) 1951 test target (2.19 µm/line) (Supplementary Fig. S2B). Further description and technical characterization of the FluoroPi device are provided in the Supplementary Information.

### Comparison of FluoroPi to a Commercial Widefield Fluorescence Microscope

**Figure 2.**
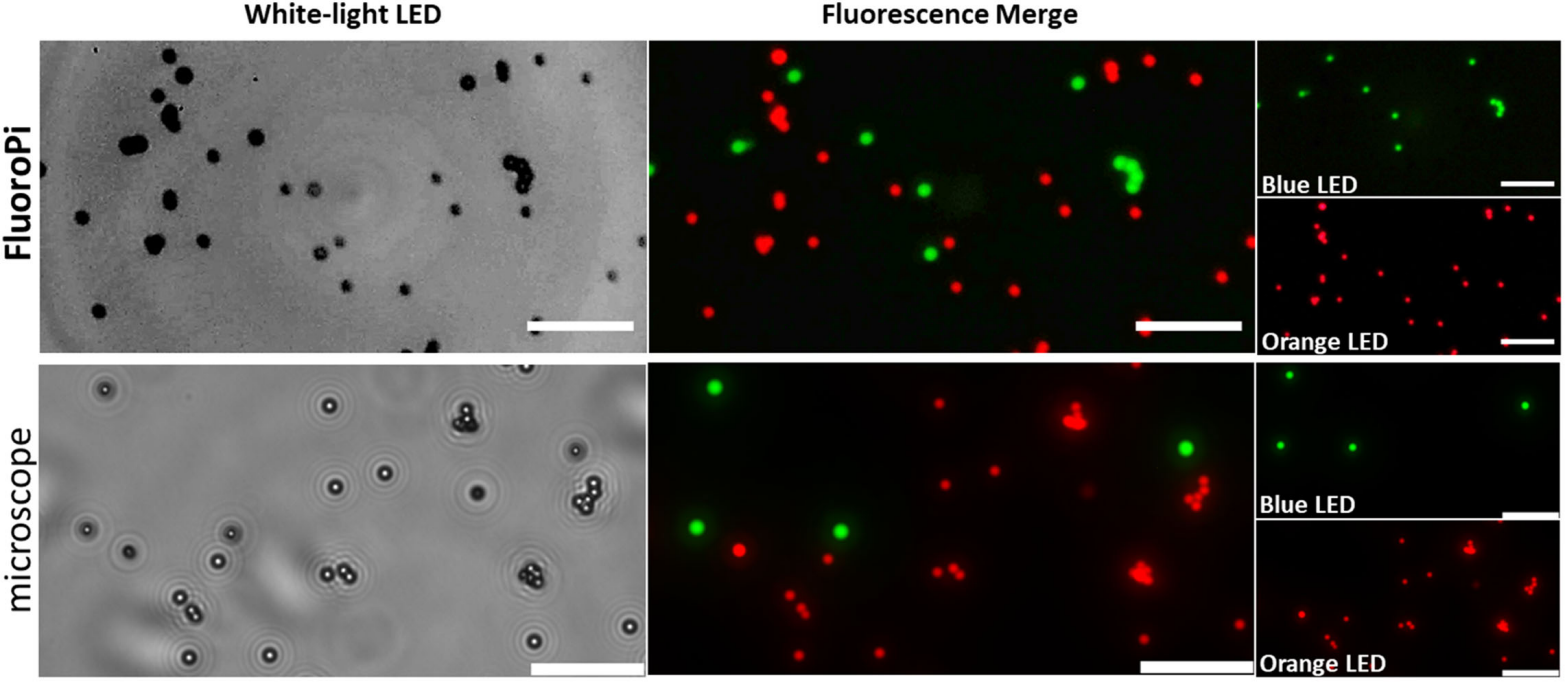
Comparison of imaging performance of FluoroPi (*top*) and commercial fluorescence microscope (*bottom*) in bright-field and fluorescence imaging modes (merged image and individual bands shown). A blue LED was used to excite the green beads, and an orange LED was used to excite the red beads. *Scale bar*: 20 µm.

In the first instance, a combination of commercial green and red microsphere beads were imaged with FluoroPi and a commercial fluorescence microscope. The commercial beads were selected for the initial validations based on their comparable size to bacteria (<2.5 µm) and similar spectral profiles to NBD-PMX (green beads) and Merocy-Van (red beads), in addition to their exhibiting high photostability and fluorescence intensity. FluoroPi was able to resolve individual beads in bright-field (white-light LED) and distinguish the two types based on their fluorescence intensity and spectral profiles (Fig. 2). There was no observed bleed-through of fluorescence, demonstrating that the filter selection was appropriate for the intended use and that there would be no requirement for spectral unmixing. The imaging performance of FluoroPi was comparable to that of the commercial widefield microscope.

**Figure 3.**
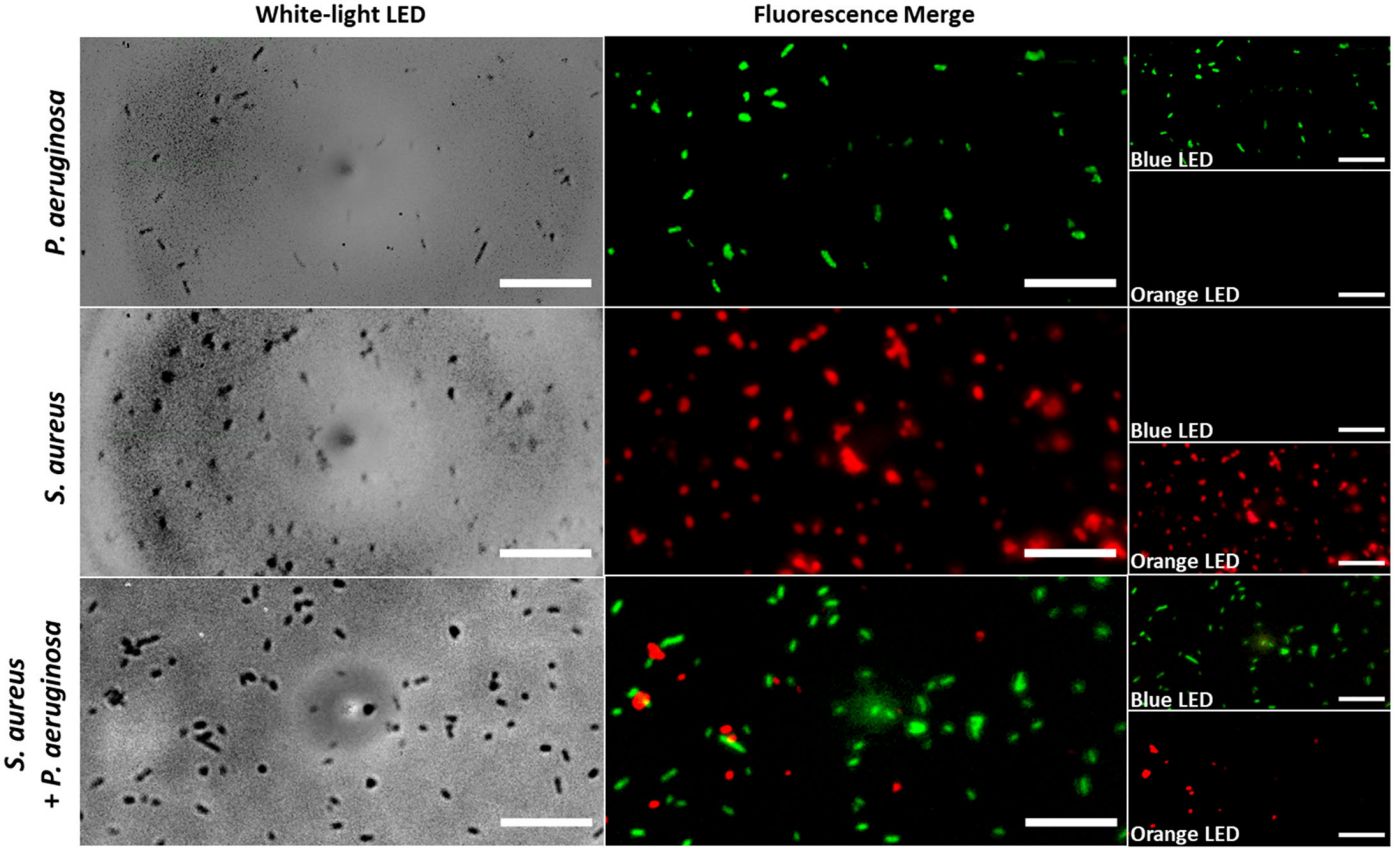
Bright-field and multiplexed fluorescence FluoroPi imaging of *P. aeruginosa* and *S. aureus* in the presence of NBD-PMX (*green*) and Merocy-Van (*red*). Imaging was conducted without removal of excess SmartProbe with sequential illumination with blue and orange LEDs across the same FoVs. Merged fluorescence images and individual bands are shown. A blue LED was used to excite NBD-PMX, and an orange LED was used to excite Merocy-Van. *Scale bar*: 20 µm. Representative FoVs are shown (*n* = 3 independent repeats).

Next, the performance of FluoroPi was assessed using *P. aeruginosa* and *S. aureus* as representative Gram-negative and Gram-positive bacteria labeled with SmartProbes NBD-PMX and Merocy-Van, respectively, and compared to the commercial microscope. The SmartProbes were imaged singularly (Supplementary Fig. S3) and multiplexed together (Fig. 3, Supplementary Fig. S4). Individual bacteria were resolved in both white-light and fluorescence imaging. As anticipated, NBD-PMX labeled only Gram-negative *P. aeruginosa* (green) and Merocy-Van labeled only Gram-positive *S. aureus* (red),^23^^,^^24^ and the two fluorescent signatures could be individually resolved from dual-probe images, demonstrating that the SmartProbes could be multiplexed and imaged, importantly, without requiring a wash step. Furthermore, the limit of detection was shown to be 10^3^ CFU/mL for *P. aeruginosa* labeled with NBD-PMX and 10^4^ CFU/mL for Merocy-Van-labeled *S. aureus* with FluoroPi (Supplementary Fig. S5), which was non-inferior to the commercial microscope (Supplementary Fig. S6).

### FluoroPi and SmartProbe Interrogation of Ex Vivo Porcine Model of MK

**Figure 4.**
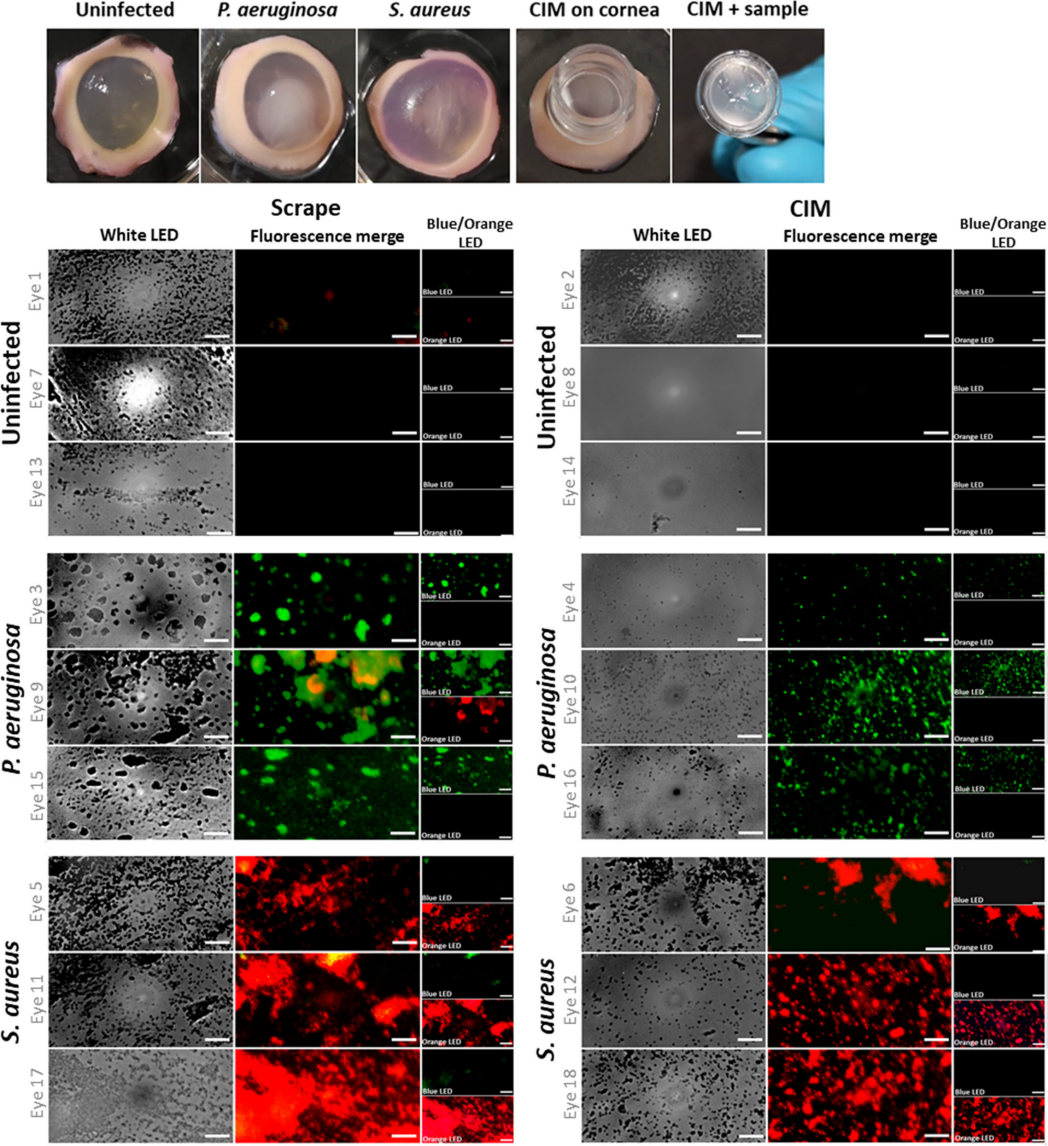
(**A**) Photograph of ex vivo porcine cornea with MK infections and sampling with the CIM collection tool. (**B**, **C**) Bright-field and multiplexed fluorescence FluoroPi imaging of scrapes (**B**) and CIMs (**C**) collected from infected ex vivo porcine cornea and transferred to glass slides in the presence of NBD-PMX (*green*) and Merocy-Van (*red*). Imaging was conducted without removal of excess SmartProbe, with sequential illumination with blue and orange LEDs across the same FoVs. Merged fluorescence images and individual bands are shown. A blue LED was used to excite NBD-PMX, and an orange LED was used to excite Merocy-Van. *Scale bar*: 20 µm. Representative FoVs are shown.

Ex vivo porcine models of MK were established with *P. aeruginosa* (*n* = 6) and *S. aureus* (*n* = 6) to represent two common bacterial pathogens isolated from clinical cases of MK.^9^ Uninfected cornea controls (*n* = 6) were subject to the same epithelium damage as the MK cases (Fig. 4a). Sampling was by routine scrape or CIM. A combined solution of NBD-PMX and Merocy-Van was added to each slide, and imaging was conducted with FluoroPi (Fig. 4) and a commercial microscope (Supplementary Fig. S7) without any additional wash steps.

White LED imaging of the uninfected control cornea scrapes (*n* = 3) demonstrated a large amount of debris deposited onto the slides. Although the size and morphology were similar to that observed for bacteria, no fluorescence signal was detected from either SmartProbe by FluoroPi or the commercial microscope (Fig. 4b, Supplementary Fig. S7a). This is in contrast to the *P. aeruginosa* (*n* = 3) and *S. aureus* (*n* = 3) MK cornea scrapes, which showed bright green (*P. aeruginosa*) and red (*S. aureus*) fluorescence across the FoVs, indicating that the Gram specificity of NBD-PMX and Merocy-Van were retained (Fig. 4b). Areas with material in the white LED image but no fluorescence image can be characterized as cellular debris. One eye infected with *P. aeruginosa* did demonstrate some off-target red fluorescence signal, detected by FluoroPi imaging across all three FoVs. This was not apparent when imaging a scrape from the same eye with the commercial microscope. The off-target signal here is most likely caused by some non-specific off-target activation of Merocy-Van within the large clumps of bacteria, which may have an altered microenvironment and favorable conditions to switch on the fluorophore. Importantly, all of the red fluorescent signal colocalized within regions of the green fluorescent signal, indicating that the true presence of Gram-positive bacteria within the sample was unlikely. Furthermore, culturing of the ex vivo porcine corneas and CIMs following sample retrieval confirmed mono-species infections (i.e., only *P. aeruginosa* was grown from the *P. aeruginosa–*infected eyes and *S. aureus* from the *S. aureus–*infected eyes).

Sample collection by the CIMs typically demonstrated less transfer of cellular debris to the slide (Fig. 4c), captured by white LED imaging of uninfected controls. The bacteria from the infected corneas were transferred from the CIMs to the slides in smaller clumps compared to scrapes. They were detectable by fluorescence imaging across all FoVs, and no off-target fluorescence was detected by FluoroPi imaging. The corneal samples retrieved by CIM were more evenly dispersed on the slides compared to the scraped samples, and they afforded greater imaging consistency between experimental replicates.

## Discussion

Fluorescence microscopy is an increasingly attractive approach for the rapid detection of pathogens^29^^,^^30^ and has greatly aided the identification of *Mycobacterium* spp*.* during smear microscopy when coupled with fluorescent imaging reporters, improving both sensitivity and specificity compared to white-light imaging with colored dyes (synonymous with traditional Gram stain in MK).^31^ Various pathogen-specific, wash-free fluorescent probes have now been reported^23^^,^^24^^,^^32^^–^^34^ that could be useful for identifying pathogens causing MK. We have previously demonstrated that NBD-PMX outperforms Gram stain in identifying Gram-negative bacteria from 160 MK patient corneal scrapes (with statistically improved sensitivity, specificity, negative predictive value, and overall accuracy).^7^ However, there is a limited availability of low-cost, simple-to-use imaging systems that are required to detect the imaging probe signal.

Typically, the development of such microscopes has been hampered by the costs associated with components such as the objectives required for submicron imaging resolution for single bacterial cell detection, as well as the availability of sensors with the required sensitivity. Here, we were able to overcome these limitations with a single mass-produced lens combined with efficient optical filtering, and we exploited recent advances in sensor technology^35^ to enable a multicolor LED-based imaging system offering submicron resolution. Moreover, the design of the FluoroPi, with its easily interchangeable LEDs and filters, means that the system is flexible and is readily modifiable should optical reporters with different spectral characteristics be required to image additional targets (such as host response biomarkers or specific bacterial or fungal species).

The combination of dual imaging modalities, white light transmission imaging, and multicolor fluorescence imaging provides a powerful platform for validation of acquired imagery, with the co-registration of morphological and fluorescence signatures helping to reduce false-positive results due to any off-target labeling or non-fluorescent cellular debris. We have demonstrated that FluoroPi performs as well as a commercially available fluorescence microscope (which is at least an order of magnitude more expensive) and is able to resolve single bacteria along with those in aggregates from ex vivo porcine models of MK (a commonly utilized model of bacterial MK)^27^^,^^36^^–^^39^ using two complementary SmartProbes to distinguish Gram-negative bacteria (NBD-PMX, green) from Gram-positive bacteria (Merocy-Van, red). The SmartProbe imaging limit of detection with FluoroPi for the exemplar Gram-negative and Gram-positive bacteria was as low as 10^3^ CFU/mL for *P. aeruginosa* labeled with NBD-PMX and 10^4^ CFU/mL for Merocy-Van-labeled *S. aureus.* Demonstrating this level of sensitivity is an important metric for characterizing FluoroPi performance because often 10^4^ to 10^5^ CFU/mL is a clinically relevant threshold for diagnosing an infection (vs. colonization) in a range of indications.^40^^,^^41^ Although MK diagnosis is not characterized in these terms, a high level of sensitivity is important clinically because the diagnostic slides often have very low numbers of bacteria present,^9^^,^^42^ and a single bacteria within a FoV can indicate a positive diagnosis. Importantly, we have shown that single bacteria within a FoV can be readily identified with FluoroPi. However, further studies with a broader panel of Gram-negative and Gram-positive MK isolates is warranted to confirm the generalizability of the limits of detection presented within this study.

We utilized our ex vivo porcine MK models to compare scrape and CIM sampling methodologies and assess the performance of the SmartProbes with the added challenge of tissue and cellular debris, a common feature of MK smear microscopy. The two SmartProbes were added in combination to freshly prepared slide samples; the coverslips were added, and the samples were imaged within the same day. No washing or additional preparation steps were required, offering ease of workflow. Overall, much more tissue debris was transferred to the slides from scrapes compared to the CIM sampling technique. This tissue debris was only visible by white-light imaging and was not stained erroneously by the SmartProbes, confirming their specificity to Gram-positive and Gram-negative bacteria.

Interestingly, the samples collected by scrape were in much larger aggregates compared to those collected by CIM. This could be attributed to the CIM being a less invasive collection technique compared to the scrape with a needle or could be attributed to the addition of saline to retrieve the sample from the CIM. Importantly, the samples collected from the ex vivo porcine corneas and labeled with NBD-PMX looked similar (including bacterial cell density within the FoV) to those collected from patients in a previous clinical study assessing the feasibility of the SmartProbe to label Gram-negative bacteria,^7^ suggesting that our model and methodology are a clinically relevant pre-clinical approach for validating our proof-of-concept FluoroPi. Moreover, the successful bacterial retrieval and SmartProbe labeling using the CIM suggest that this could be employed as a less-invasive sampling technique for this diagnostic strategy and could open opportunities for sample collection and point-of-care diagnosis within primary and secondary care or facilities without dedicated microbiology laboratory infrastructure, a major limitation and barrier to care currently for patients with MK.

To translate the FluoroPi technology, we are adapting the current prototype to a more robust, user-friendly version. This will include an on-device graphical user interface for better ease of operation, integration of the current postprocessing to enable real-time image processing, and full enclosure of the system to make it more robust. Three-dimensional printing of the enclosure, mountings, and stages, along with the use of a single power supply and LED driver (which are becoming available for the Raspberry Pi), will further greatly reduce the cost of the current version. The next iteration of the FluoroPi device will then undergo evaluation with clinical samples as part of future validation studies.

## Conclusions

We have developed and evaluated a low-cost accessible imaging platform capable of multicolor fluorescence, combined with white-light morphological imaging. The device has been shown to have the resolution to resolve single bacteria and, in conjunction with appropriate fluorescent reporters, successfully distinguish between the exemplar Gram-negative and Gram-positive bacteria at the cost point of a cell phone. We have demonstrated the feasibility of bacterial keratitis smear microscopy in an ex vivo porcine MK infection model with FluoroPi, with slides prepared by both conventional corneal scrape, and a less invasive approach with CIM for sample retrieval. The Gram statuses of the model bacteria (*P. aeruginosa* and *S. aureus*) were successfully identified across all corneas evaluated by both sample collection techniques. The sample preparation methodology, image capture, and postprocessing are straightforward and could be performed across multiple levels of the healthcare system. Our prototype FluoroPi and optical SmartProbes demonstrate that our approach is sensitive (able to detect single bacteria) and specific for bacterial Gram status detection.

## Supplementary Material

Supplement 1
