# Supplementary material for: FluoroPi Device With SmartProbes: A Frugal Point-of-Care System for Fluorescent Detection of Bacteria From a Pre-Clinical Model of Microbial Keratitis
Source: Transl Vis Sci Technol. 2023 Jul 3;12(7):1. doi: 10.1167/tvst.12.7.1 (PMC10324419; doi:10.1167/tvst.12.7.1)
Supplement: Supplement 1 [file tvst-12-7-1_s001.pdf]

<u>Supplementary Information</u>

FluoroPi device with SmartProbes: a frugal point-of-care system for fluorescent detection of bacteria from a pre-clinical model of microbial keratitis

Syam Mohan P.C. Mohanan[1]*, Kay Russell[1]*, Sheelagh Duncan [1], Alex Kiang[1], Charles Lochenie[1], Emma Duffy[1], Stephnie Kennedy[2], N. Venkatesh Prajna[3], Rachel L. Williams[2], Kevin Dhaliwal[1], Gareth O.S. Williams[1#] and Bethany Mills[1#]

[1] Translational Healthcare Technologies Group, Centre for Inflammation Research, University of Edinburgh, Edinburgh, UK

[2]Department of Eye and Vision Science, University of Liverpool, Liverpool, UK

[3] Department of Cornea and Refractive Surgery, Aravind Eye Hospital, Madurai, India

*[#]Authors contributed equally

E-mail: G.O.S.Williams@ed.ac.uk, Beth.Mills@ed.ac.uk

Supplementary Materials and Methods

FluoroPi Instrumentation

The FluoroPi device consists two main modules; a detection module containing a camera and associated lenses for image capture and an illumination module consisting of multiple LEDs and lenses for sample illumination and excitation. There is a microscopy slide holder for sample placement and adjustment, and a Raspberry Pi single board computer (Version 3B+) [22] with associated touch screen for user input. The Raspberry Pi camera was connected to the single board computer through its dedicated CSI2 ribbon cable port and was used for both the fluorescence and white-light image acquisition.

The illumination module comprised of blue (470 nm, M470L3 Thorlabs) and orange (590 nm, M590L4 Thorlabs) excitation LEDs for fluorescence imaging. White-light transmission imaging of the sample was performed using a white-light emitting through hole LED (C503D-WAN-CCbEb151, Cree). 30 mm focal length lenses (LA1289-A-MLThorlabs) were connected to the distal end of the lens tubes attached to the LEDs. These lenses were used to produce a 1 mm spot, filling the system's field of view (FoV). The blue and orange LEDs had optical powers of 4 and 2.28 mW respectively, measured at the sample plane after focusing, using a Thorlabs power meter (PM100D with S130C Sensor). The optical output power of white LED was measured to be 23 μW by setting the power meter to 520 nm assuming low deviation due to the spectral response of the sensor.

The image detection module consisted of a 12-megapixel CMOS camera (RPI-HQ-CAMERA) coupled with a low-cost primary lens (Raspberry Pi camera V2 Lens) using a half inch lens tube (SM05L30 Thorlabs). The primary lens of 3.6 mm focal length was placed at the distal end of the lens tube to collect the fluorescence signal from the sample. An emission filter appropriate for removing any remaining excitation light (ET470/625/780M for the blue LED, ET610LP for the orange LED, (Chroma)) was placed in between the camera sensor and the lens. Note that the multiband filter used for the removal of the blue LED has a second transmission band that allows for partial transmission of

the emission from the Merocy-Van SmartProbe (Figure 1C), the full filter transmission profile is given in Supplementary Figure S1. Future version of the device will optimise filters for out of band rejection.

A photograph of the proof-of-concept prototype device is shown in Figure 1B. An X-Y stage with a microscopy slide holder (XFY1, Thorlabs) was incorporated to support the sample during the experiment. The choice of lenses produced an optical magnification of ~25x providing sufficient resolution for the target applications.

Determination of FluoroPi field of view and imaging resolution.

The imaging FoV was determined using a haemocytometer (Fuchs Rosenthal C-Chip Disposable Haemocytometer, LabTech) mounted within the slide holder and white-light LED imaging of the etched grid pattern with FluoroPi (Supplementary Figure S2A). The distance between grid-lines was determined by imaging the same slide on a calibrated wide-field microscope (Leica DMi8 Basic) and measuring the distance between grid lines using LAS X software (Leica) (Supplementary Figure S2A). The calculated distances were incorporated with Image J (Fiji) to determine the FoV and set scale bars for subsequent data collected with FluoroPi. The imaging resolution of FluoroPi was determined using a United States Air Force (USAF) 1951 test target illuminated with white-light. The smallest available lines (group 7, elements 1-6) of the test target were very clearly captured (Supplementary Figure S2B) by FluoroPi, and were easily resolvable. To calculate the absolute resolution of the imaging platform, a cross sectional line profile was taken across the smallest elements (2.19 µm in width) in the target (group 7 element 6, Supplementary Figure S2B4). Assuming the feature edges on the target are infinitely steep the resolution of the system can be determined by a Gaussian fit to the profile of the feature edge. This fit yielded an absolute resolution of the imaging platform of ~630 nm.

FluoroPi Image Processing

Image processing was conducted with Image J (Fiji) software. The use of simple lenses and filters led to uneven sample illumination causing significant spatial artefacts along with the presence of scattering of light. To correct for these artefacts and improve image quality, a background slide image was

subtracted from corresponding sample during post-processing. Resultant images were subsequently brightness and contrast enhanced. The same enhancement was applied to all images within a sample type to ensure consistency and the ability to cross compare.

Ex vivo porcine cornea preparation

Fresh porcine eyes were received from "pre-tank" pigs within 12 h of their slaughter from a local abattoir (Browns Food Group (Quality Pork Processors Ltd.)). The eyes were kept on ice during transportation and at 4 °C thereafter. Any remaining extra-ocular tissue was removed, and the eyes were stored at in phosphate buffered saline (PBS) (Invitrogen) overnight. The eyes were immersed in PBS containing 1% [v/v] penicillin/streptomycin and amphotericin B (P/S/AmpB) (Merck, UK) for 2 min, followed by 2 min in 3 % [v/v] povidone iodine (Videne, UK) diluted in PBS. The eyes were washed a further three times in antibiotic-free PBS.

Next, the epithelium was removed from the cornea mechanically through scraping with a scalpel blade. The cornea-scleral rims were dissected from the globe, and the lens, ciliary body, iris and trabecular meshwork removed. The cornea-scleral rims were submerged again for 2 min each in PBS:P/S/AmpB, followed by the iodine solution and then washed thrice in PBS.

The internal side of the cornea-scleral rims were filled with 1 % [w/v] ultra-pure-agarose dissolved into Dulbecco's Modified Eagles Medium (DMEM, ThermoFisher) containing Foetal Bovine Serum 10% [v/v] (Labteck, UK). The agarose supports were set at room temperature, and the cornea-scleral rims were placed into individual wells of a 6-well plate (Corning) epithelial side up.

Supplementary Figures

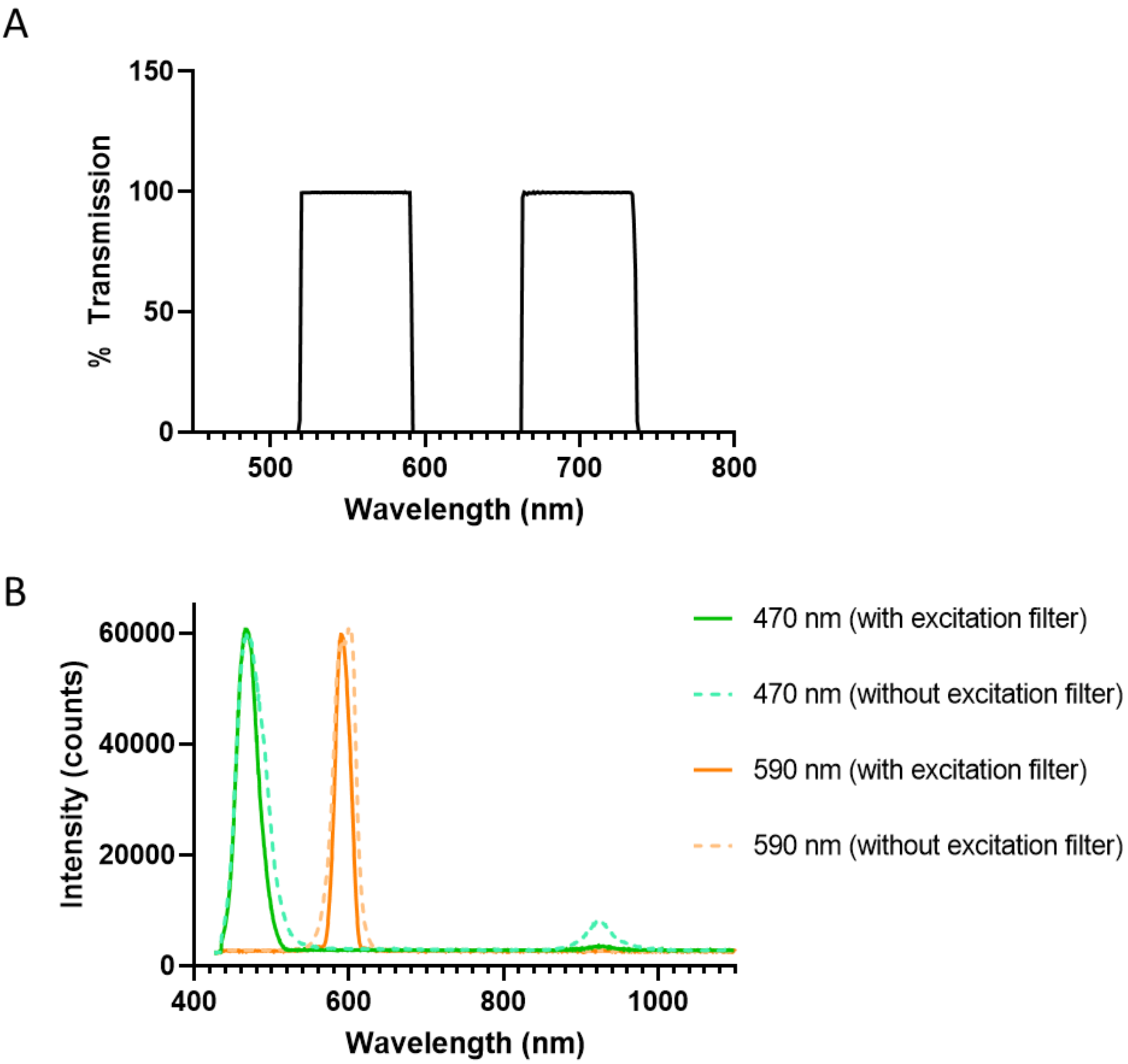

ESI Figure S1. (A) Transmission profile of the filter used in conjunction with the blue excitation LED, note that the second transmission window partially overlaps with the emission spectrum of the Merocy-Vanc SmartProbe (Figure 1C). **(B)** Excitation spectra of 470 nm and 590 nm LEDs with and without excitation filter.

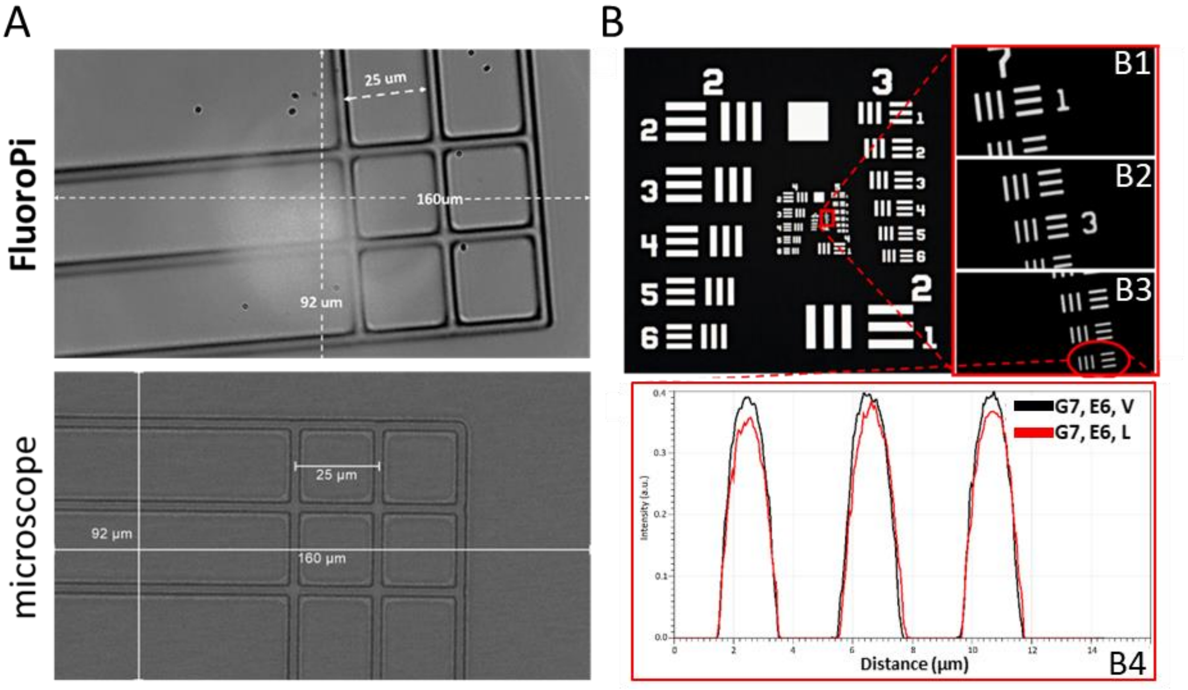

ESI Figure S2. Characterisation of FluoroPi FoV and imaging resolution. (**A**) Imaging of haemocytometer slide with FluoroPi (top) and commercial microscope (bottom). (**B**) USAF 1951 test target used to determine imaging resolution of FluoroPi. (**B1-3**) Images of USAF target group 7 captured with FluoroPi. (**B4**) Cross sectional profiles of lateral and vertical lines of the smallest elements of USAF target group 7.

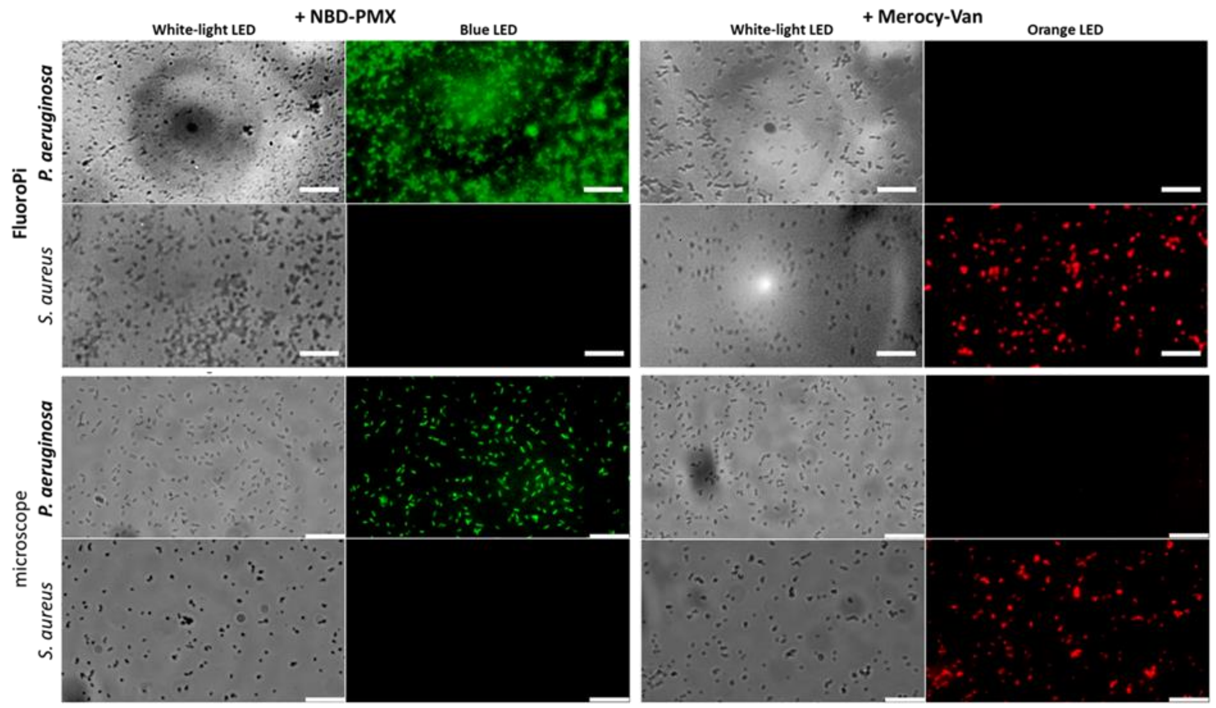

ESI Figure S3. Bright-field and single-channel fluorescence FluoroPi imaging of *P. aeruginosa* and *S. aureus* in the presence of NBD-PMX (green) or Merocy-Van (red). Imaging was conducted without removal of excess SmartProbe. The Blue LED was used to excite NBD-PMX, and the Orange LED was used to excite Merocy-Van. Top: FluoroPi imaging. Bottom: commercial microscope imaging with FITC (green) and Texas Red (red) imaging parameters. Scale bar = 20 µm. Representative FoVs shown , n = 3 independent repeats.

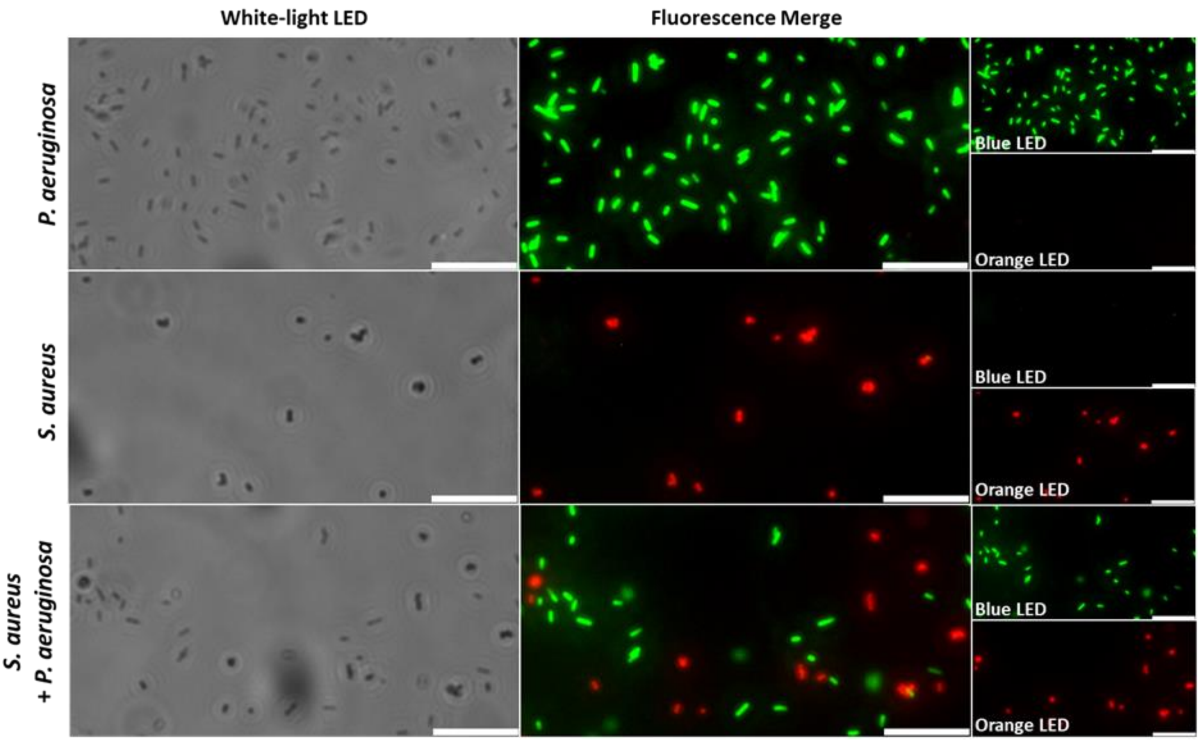

ESI Figure S4. Bright-field and dual-channel fluorescence commercial microscope imaging of *P. aeruginosa* and *S. aureus* in the presence of NBD-PMX (green) and Merocy-Van (red). Imaging was conducted without removal of excess SmartProbe. Imaging with FITC (green) and Texas Red (red) imaging parameters. Merged fluorescence image and individual bands shown. Scale bar = 20 µm. Representative FoVs shown , n = 3 independent repeats.

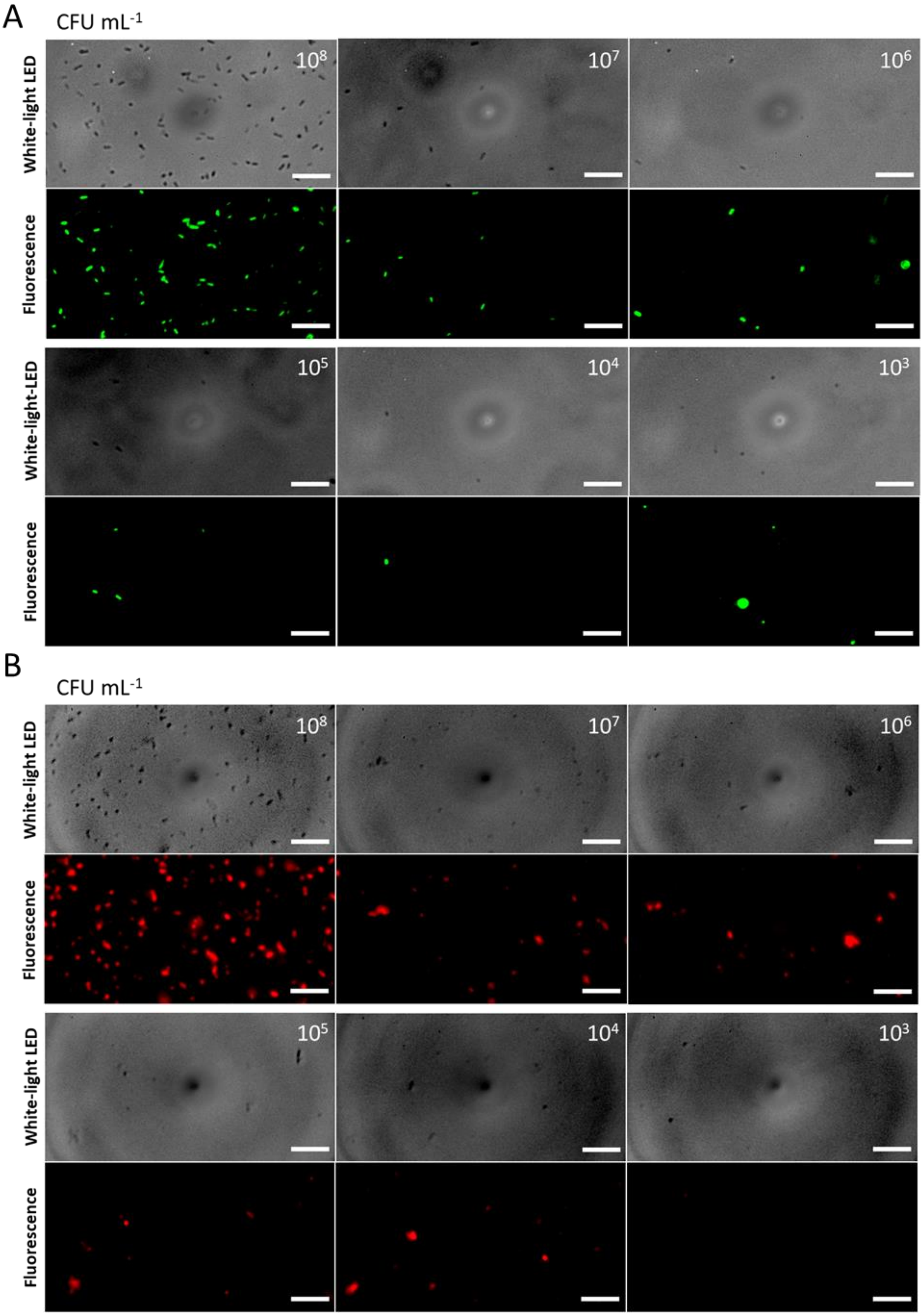

ESI Figure S5. FluoroPi **b**right-field and single-channel fluorescence FluoroPi imaging of serial dilutions of (**A**) *P. aeruginosa* with NBD-PMX (green) and (**B**) *S. aureus* with Merocy-Van (red) to determine bacterial limit of detection. Imaging was conducted without removal of excess SmartProbe. The Blue LED was used to excite NBD-PMX, and the Orange LED was used to excite Merocy-Van. Scale bar = 20 µm. Representative FoVs shown, n = 3 independent repeats.

107

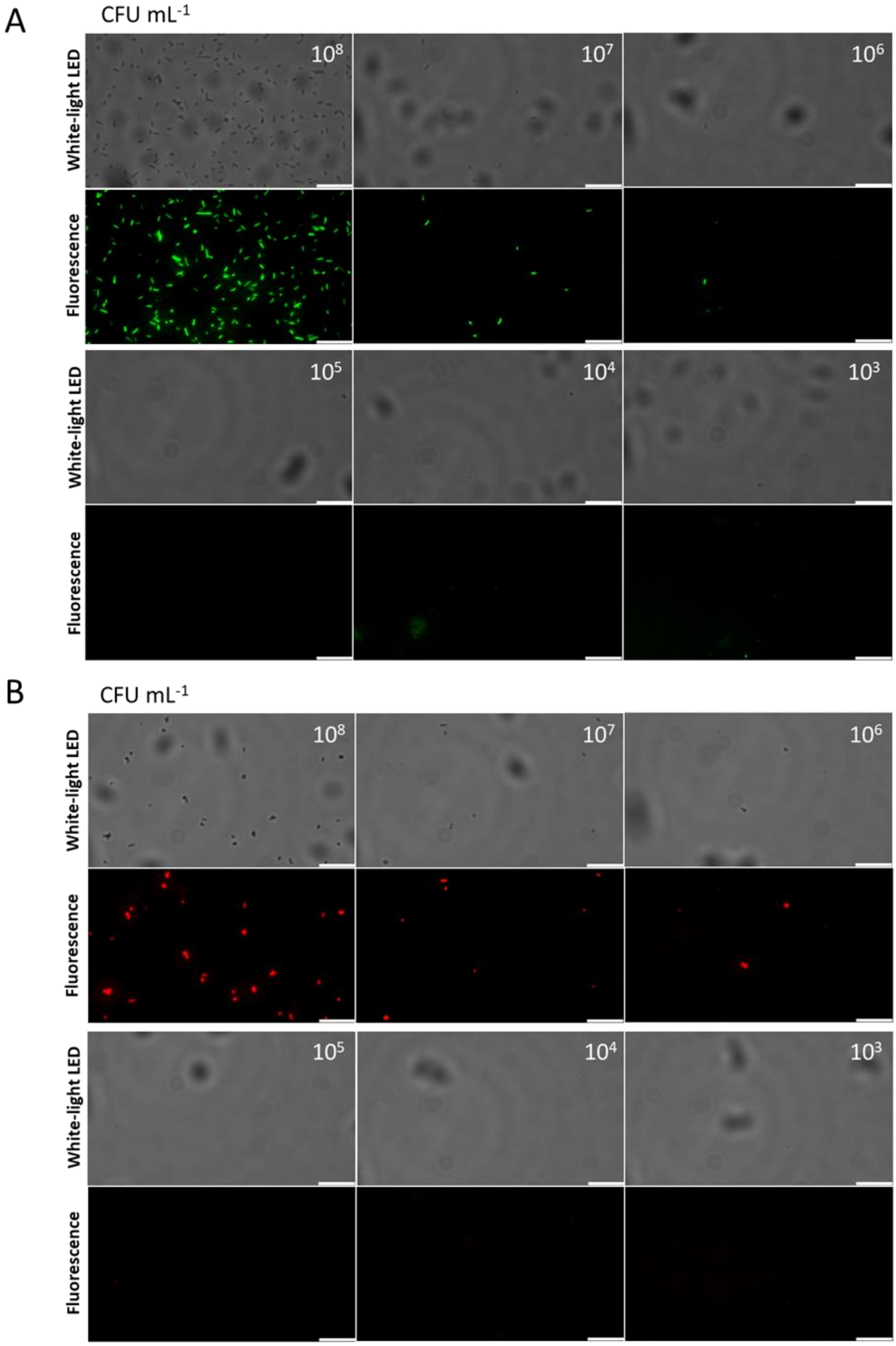

ESI Figure S6. Commercial microscope bright-field and single-channel fluorescence microscope imaging of serial dilutions of (**A**) *P. aeruginosa* with NBD-PMX (green) and (**B**) *S. aureus* with Merocy-Van (red) to determine bacterial limit of detection. Imaging was conducted without removal of excess SmartProbe. Imaging with (A) FITC (green) and (B) Texas Red (red) imaging parameters. Scale bar = 20 µm. Representative FoVs shown, n = 3 independent repeats.

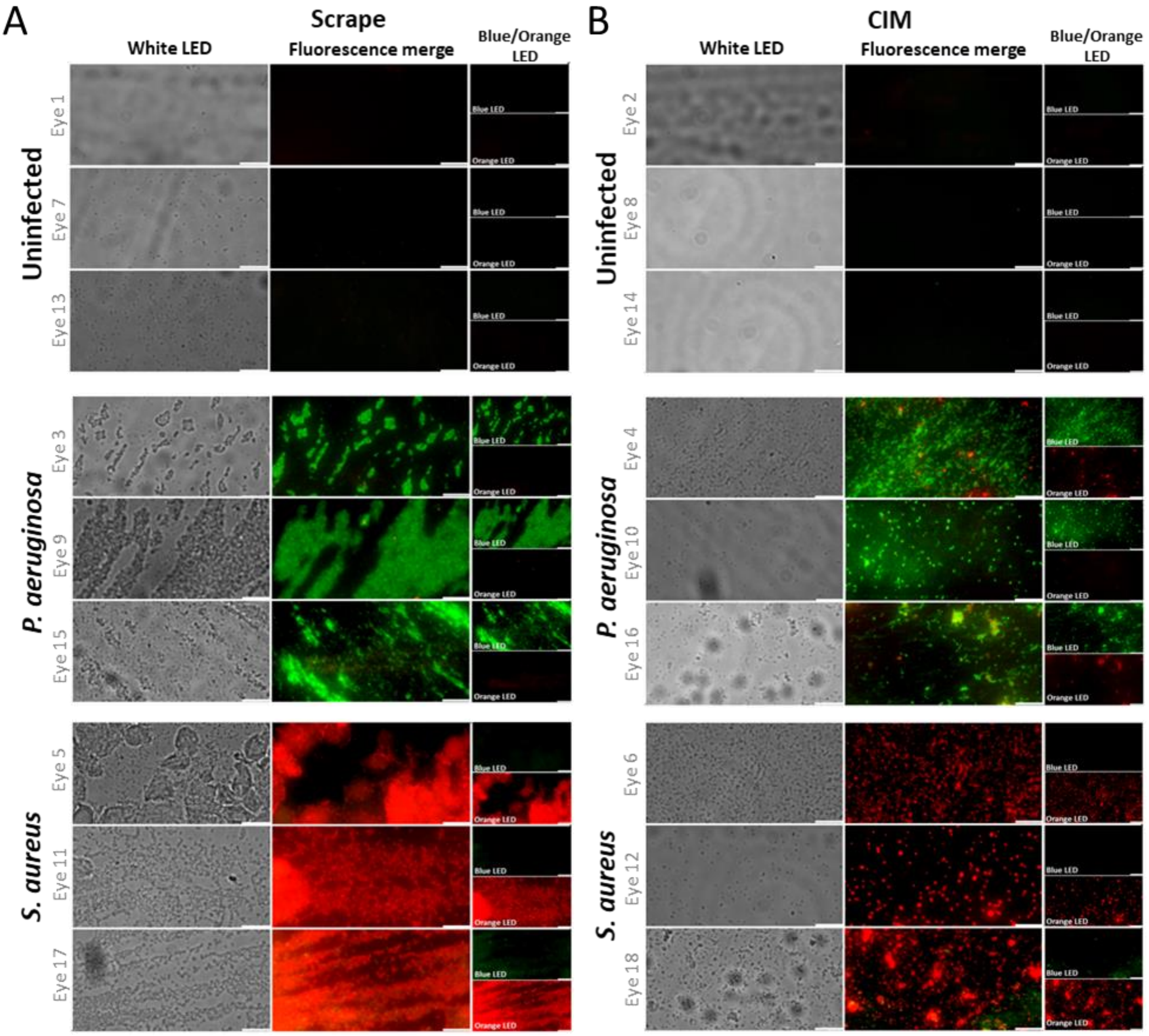

ESI Figure S7. Bright-field and multiplexed fluorescence imaging of **(A)** scrapes and **(B)** CIMs collected from infected *ex vivo* porcine cornea in the presence of NBD-PMX (green) and Merocy-Van (red). Imaging was conducted with commercial microscope without removal of excess SmartProbe. Merged fluorescence image and individual bands shown. Imaging with FITC (green) and (B) Texas Red (red) imaging parameters. Scale bar = 20 µm. Representative FoVs shown.
